# Subgroups of Prediabetes and the Risk of Cardiometabolic Multimorbidity in Chinese Adults: A Prospective Cohort Study

**DOI:** 10.1111/1753-0407.70224

**Published:** 2026-04-15

**Authors:** Yan Liu, Miao He, Yu Liu, Zilin Sun, Shanhu Qiu

**Affiliations:** ^1^ Department of Endocrinology Zhongda Hospital, Institute of Diabetes, School of Medicine, Southeast University Nanjing China; ^2^ Department of General Practice Zhongda Hospital, Institute of Diabetes, School of Medicine, Southeast University Nanjing China

To the Editor,

1

Cardiometabolic multimorbidity (CMM), defined as the coexistence of two or more cardiometabolic diseases including heart diseases, stroke, and diabetes, represents a growing global health challenge, particularly among aging populations [1, 2, 3]. Previous studies have shown that prediabetes, a heterogeneous metabolic condition, is associated with higher risks of individual diseases within the CMM spectrum [4, 5]. However, it remains unclear whether data‐driven, cluster‐based subgroups of prediabetes derived from commonly measured clinical parameters are associated with the risk of developing CMM. We conducted this study based on a large prospective Chinese cohort survey to assess the association.

Participants were from the China Health and Retirement Longitudinal Study, a nationwide survey of adults aged ≥ 45 years in 28 provinces of China [6]. Of the 3622 participants with prediabetes at baseline, cluster analysis for prediabetes grouping was performed using k‐means clustering, based on age, body mass index (BMI), glycated hemoglobin (HbA1c), and triglyceride‐glucose (TyG) index [7]. After excluding participants with prevalent CMM at baseline or missing outcome data, 3132 participants who were followed‐up for about 4 years were analyzed. Prediabetes was defined as fasting plasma glucose (FPG) of 5.6–6.9 mmol/L or HbA1c of 5.7%–6.4% [8]. Diabetes was diagnosed as FPG ≥ 7.0 mmol/L, HbA1c ≥ 6.5%, self‐reported diagnosis, or use of glucose‐lowering medication [8].

Three subgroups of prediabetes were identified (Figure S1). Of the included 3622 participants for the cluster analysis, 1310 (36.2%) was clarified as cluster 0, 1206 (33.3%) was as cluster 1, and 1106 (30.5%) was as cluster 2. Cluster 0 was characterized by youngest age and lowest HbA1c, cluster 1 was by the highest TyG and BMI, and cluster 2 was by the lowest BMI and the oldest age. During 4‐year follow‐up, 116 participants developed incident CMM (Table 1). The cumulative incidence of CMM was highest in cluster 1 (5.5%), followed by cluster 2 (3.9%) and cluster 0 (1.9%). Compared with cluster 0, both cluster 1 and cluster 2 exhibited a markedly increased risk of CMM (adjusted OR 2.71, 95% CI 1.58–4.66, and 1.91, 95% CI 1.08–3.38; respectively). The associations were consistent across different demographic subgroups and remained robust in sensitivity analyses (Figure S2 and Table S1).

**TABLE 1:** The association between different clusters and CMM (*N* = 3132).

	No. of events/Total *N* (%)	Model 1^a^	Model 2^b^	Model 3^c^
		OR (95% CI)	OR (95% CI)	OR (95% CI)
CMM				
Cluster 0	22/1145 (1.9)	1 (ref)	1 (ref)	1 (ref)
Cluster 1	57/1042 (5.5)	2.95 (1.79, 4.87)	2.82 (1.71, 4.66)	2.71 (1.58, 4.66)
Cluster 2	37/945 (3.9)	2.08 (1.22, 3.55)	1.96 (1.13, 3.40)	1.91 (1.08, 3.38)

*Note:* OR, odds ratio; CI, confidence interval.

^a^ Model 1: crude model.

^b^ Model 2: adjusted by sex, marital status, educational level and residence.

^c^ Model 3: adjusted by sex, marital status, educational level, residence, smoking status, drinking status, systolic blood pressure, C‐reactive protein, total cholesterol, low‐density lipoprotein cholesterol, cardiovascular disease.

Prior studies have employed similar approaches to characterize the heterogeneity of prediabetes by cluster‐analysis based on parameters, such as polygenic risk scores and magnetic resonance derived markers. However, they were not commonly measured in clinical routine [9, 10, 11]. In contrast, our cluster analysis was based on four commonly measured variables. We found that participants characterized by higher BMI and greater insulin resistance (cluster 1) had the highest risk for CMM compared with cluster 0, supporting the role of adiposity and insulin resistance in the development of cardiometabolic dysfunction [12, 13]. Moreover, participants characterized by the oldest age and the lowest BMI (cluster 2) also showed an increased risk, indicating that aging might be an independent risk factor for CMM [14]. These findings, taken together, highlight the heterogeneity of prediabetes and suggest that risk stratification based on metabolic phenotypes may provide novel opportunities for early, phenotype‐guided precision interventions.

## Author Contributions

Conception and design of the study: Yan Liu, Shanhu Qiu, and Zilin Sun. Acquisition, analysis, or interpretation of data: Yan Liu, Yu Liu, Miao He, Shanhu Qiu, and Zilin Sun. Writing and modifying of the manuscript: Yan Liu, Shanhu Qiu, and Zilin Sun. Study supervision: Shanhu Qiu and Zilin Sun. All authors reviewed and approved the final version of the article submitted for publication.

## Funding

This study was partly supported by the Noncommunicable Chronic Diseases‐National Science and Technology Major Project (grant No. 2023ZD0509000), the National Natural Science Foundation of China (grant No. 72374043), the Key Research and Development Program in Jiangsu Province (BE2022828), and the Chronic Disease Management Research Project of National Health Commission Capacity Building and Continuing Education Center (grant No. GWJJMB202510024098). Yan Liu is supported by China Scholarship Council (CSC). The funders had no roles in the design of the study and collection, analysis, and interpretation of data or in writing the manuscript.

## Ethics Statement

China Health and Retirement Longitudinal Study was approved by the Institutional Review Board of Peking University (approval number: IRB00001052‐11015 for the household survey and IRB00001052‐11014 for blood samples), and all participants provided written informed consent.

## Conflicts of Interest

The authors declare no conflicts of interest.

## Supporting information


**Data S1:** Supporting Information.
**Figure S1:** Characteristics of clusters and distribution of participants. T‐SNE visualization of participant data (A), box plot of cluster variables by cluster (B) and frequency distribution of clusters (C). T‐SNE, t‐distribution stochastic neighborhood embedding; BMI, body mass index; TyG, triglyceride and glucose index; HbA1c, glycated hemoglobin.
**Figure S2:** Subgroup analysis of different clusters and CMM. The model was adjusted for sex, marital status, educational level, residence, smoking status, drinking status, systolic blood pressure, C‐reactive protein, total cholesterol, low‐density lipoprotein cholesterol, cardiovascular disease. CMM, cardiometabolic multimorbidity; OR, odds ratio; CI, confidence interval.
**Table S1:** The sensitivity analyses of different clusters and CMM.

## Data Availability

The data that support the findings of this study are available on request from the corresponding author. The data are not publicly available due to privacy or ethical restrictions.
