# Supplementary material for: Subgroups of Prediabetes and the Risk of Cardiometabolic Multimorbidity in Chinese Adults: A Prospective Cohort Study
Source: J Diabetes. 2026 Apr 15;18(4):e70224. doi: 10.1111/1753-0407.70224 (PMC13080258; doi:10.1111/1753-0407.70224)
Supplement: Supplementary file 1 — Data S1: Supporting Information. Figure S1: Characteristics of clusters and distribution of participants. T‐SNE visualization of participant data (A), box plot of cluster variables by cluster (B) and frequency distribution of clusters (C). T‐SNE, t‐distribution stochastic neighborhood embedding; BMI, body mass index; TyG, triglyceride and glucose index; HbA1c, glycated hemoglobin. Figure S2: Subgroup analysis of different clusters and CMM. The model was adjusted for sex, marital status, educational level, residence, smoking status, drinking status, systolic blood pressure, C‐reactive protein, total cholesterol, low‐density lipoprotein cholesterol, cardiovascular disease. CMM, cardiometabolic multimorbidity; OR, odds ratio; CI, confidence interval. Table S1: The sensitivity analyses of different clusters and CMM. [file JDB-18-e70224-s001.docx]

**Data S1: Supporting Information**

**Figure S1. Characteristics of clusters and distribution of participants.**

T‐SNE visualization of participant data (A), box plot of cluster variables by cluster (B) and frequency distribution of clusters (C). T‐SNE, t-distribution stochastic neighborhood embedding; BMI, body mass index; TyG, triglyceride and glucose index; HbA1c, glycated hemoglobin.

**Figure S2. Subgroup analysis of different clusters and CMM.**

The model was adjusted for sex, marital status, educational level, residence, smoking status, drinking status, systolic blood pressure, C-reactive protein, total cholesterol, low-density lipoprotein cholesterol, cardiovascular disease.

CMM, cardiometabolic multimorbidity; OR, odds ratio; CI, confidence interval;

**Table S1. The sensitivity analyses of different clusters** **and CMM.**

|  | **Cases/Total N (%)** | **OR (95% CI)** |
| --- | --- | --- |
| Cluster 0 | 9/341 (2.6) | 1 (ref) |
| Cluster 1 | 37/512 (7.2) | 2.69 (1.17, 6.17) |
| Cluster 2 | 18/323 (5.6) | 2.13 (0.86, 5.26) |

Sensitivity: excluding participants with FPG < 6.10mmol/L (n = 1956). The model was adjusted for sex, marital status, educational level, residence, smoking status, drinking status, systolic blood pressure, C-reactive protein, total cholesterol, low-density lipoprotein cholesterol, cardiovascular disease. CMM, cardiometabolic multimorbidity; OR, odds ratio; CI, confidence interval;
